# Yawning as a Remedy

**Published:** 1892-12

**Authors:** 


**Yawning as a Remedy.**—According to current ideas, yawning in good society is an improper sign of weariness; according to the teachings of physiology, it is a long-drawn, forcible inspiration followed by a shorter respiration; according to Dr. Naegeli, it is one of nature's many remedies, the proper application of which depends upon good judgment.

In yawning, not only the muscles which move the lower jaw are used, but also the breathing muscles of the chest, and he who yawns to his heart's content also raises and extends the arms. In the deepest inspiration the chest remains extended for a short time, the eyes are almost or entirely closed, the ears somewhat raised, the nostrils dilated. Inside the mouth, the tongue becomes round and arched, the palate stiffly stretched, and the uvula is raised, almost entirely closing the space between the nose and throat. At the beginning of the inspiration a cracking noise is heard in the ears, a proof that the duct leading to the hearing also succumbs to this stretching.

If the yawning has reached the deepest point, it will require from one to one and a half seconds for it to become noticeable to the hearing. In order to observe this, let one place himself at a sufficient distance from a clock, so that its ticking will not be easily heard, and yawn deeply. During this deep breathing the sound of the clock is not perceptible to the most careful listening. All this simply goes to show that yawning sets a number of muscles to work, and particularly those which are not directly subject to the will.

Although one yawning does not present a very agreeable appearance, it is very agreeable to himself, for the stretching of the muscles causes a feeling of comfort; it acts like massage, and is the most natural gymnastics of the lungs imaginable. Dr. Naegeli, therefore, advises people not to concern themselves with so-called decency, but every morning and evening, and as often as possible, to exercise the lungs and all the muscles of respiration by yawning and stretching, as many chronic lung troubles may thus be prevented.

Dr. Naegeli orders the patient troubled with too much wax in the ear, accompanied with pain, to yawn often and deeply. The pain will soon disappear. [But what of the wax?—Ed. Tex. Med. Journal.] He also, in cases of nasal catarrh, inflammation of the palate, sore throat, and earache, orders the patient as often as possible during each day to yawn from six to ten times successively, and immediately afterward to swallow. The result will be surprising. If one looks upon yawning as a natural massage for certain organs, he will reach a satisfactory explanation of its curative properties.—*Translated for Public Opinion from the German of Mr. Julius Stinde, in the Berlin Unsere Zeit.* —*Southern Practitioner.*

